# Delayed Diagnosis of Congenital Combined Pituitary Hormone Deficiency including Severe Growth Hormone Deficiency in Children with Persistent Neonatal Hypoglycemia—Case Reports and Review

**DOI:** 10.3390/ijms231911069

**Published:** 2022-09-21

**Authors:** Joanna Smyczyńska, Natalia Pawelak, Maciej Hilczer, Andrzej Lewiński

**Affiliations:** 1 Department of Pediatrics, Endocrinology, Diabetology and Nephrology, Medical University of Lodz, 90-419 Lodz, Poland; 2 Department of Endocrinology and Metabolic Diseases, Polish Mother’s Memorial Hospital—Research Institute, 93-338 Lodz, Poland; 3 Department of Endocrinology and Metabolic Diseases, Medical University of Lodz, 90-419 Lodz, Poland

**Keywords:** growth hormone, insulin-like growth factor-1, growth hormone deficiency, combined pituitary hormone deficiency, persistent neonatal hypoglycemia, pituitary hypoplasia, pituitary stalk interruption syndrome, whole exome sequencing

## Abstract

Apart from stimulation of human growth and cell proliferation, growth hormone (GH) has pleiotropic metabolic effects in all periods of life. Severe GH deficiency is a common component of combined pituitary hormone deficiency (CPHD). CPHD may be caused by mutations in the genes encoding transcription factors and signaling molecules involved in normal pituitary development; however, often its genetic cause remains unknown. Symptoms depend on which hormone is deficient. The first symptom of GH or adrenocorticotropic hormone (ACTH) deficiency may be persistent hypoglycemia in apparently healthy newborns, which is often neglected. Diagnosing CPHD is based on decreased concentrations of hormones secreted by the anterior pituitary and peripheral endocrine glands. Findings in magnetic resonance imaging vary widely, including anterior pituitary hypoplasia/aplasia or pituitary stalk interruption syndrome (PSIS). Delayed diagnosis and treatment can be life-threatening. GH therapy is necessary to recover hypoglycemia and to improve auxological and psychomotor development. We present two girls, diagnosed and treated in our departments, in whom the diagnosis of CPHD was delayed, despite persistent neonatal hypoglycemia; and a review of similar cases, with attention paid to progress in the genetic assessments of such patients, since the introduction of whole exome sequencing that is especially important for PSIS.

## 1. Introduction

Combined pituitary hormone deficiency (CPHD) is a disease related to insufficient secretion of two or more hormones produced in the anterior pituitary [1]. The main hormones synthetized and secreted by the anterior pituitary are: growth hormone (GH), thyrotropin (TSH), gonadotropins (follicle-stimulating hormone—FSH and luteinizing hormone—LH), adrenocorticotropic hormone (ACTH) and prolactin (Prl). The posterior lobe of pituitary gland contains and secretes oxytocin and antidiuretic hormone (ADH). The main function of GH, also referred to as somatotropin, is stimulation of linear growth of children and cell proliferation. Moreover, GH’s pleiotropic effects in different cells and tissues are important in the regulation of metabolism during all periods of life. Severe GH deficiency (GHD), especially combined with ACTH deficiency, may be a cause of persistent and recurrent hypoglycemia.

Proper development and functioning of the pituitary gland require the cooperation of different genes mediating pituitary development, transcription factors and signaling molecules. Their defects may manifest as isolated hormone deficiencies or CPHD. Hypopituitarism can be a component of specific syndromes. However, in most cases, the genetic cause of congenital hypopituitarism remains uncertain [2].

In some patients, CPHD is related to anterior pituitary hypoplasia or aplasia, or a specific complex of abnormalities in the hypothalamic pituitary region which can be detected by magnetic resonance imaging (MRI). These include anterior pituitary hypoplasia, invisible pituitary stalk and ectopic posterior pituitary, which constitute pituitary stalk interruption syndrome (PSIS), and empty sella syndrome (however, the latter syndrome is not irrevocably related to hormonal deficiencies) [3,4].

## 2. Case Reports

Delayed diagnosis of congenital CPHD may be a cause of impaired somatic and psychomotor development of children. We present two patients with CPHD who were diagnosed at the ages of 3 months and 12 months, despite persistent or recurrent hypoglycemia in infancy.

### 2.1. Patient 1

A girl was delivered at the 39th week of gestation by cesarean section for obstetric indications (mother after cesarean section), with birth weight 3100 g and length 49 cm, and Apgar score 8/9/9. Birth size of the child with respect to gestational age (GA) was normal: birth weight SDS −0.77, birth length SDS −0.44, according to Niklasson et al. [5]. Her parents are healthy and not related.

According to the documentation from the neonatal department, the child after birth presented with features of immaturity, corresponding to 36th week of gestation. The girl required tactile stimulation, and temperature instability was observed. After 24 h of life, blood saturation was below 90%, and hypoglycemia was detected: the lowest glucose level was 17 mg/dL (0.94 mmol/L) in her first day of life and 34 mg/dL (1.89 mmol/L) in the next few days. It should be mentioned here that normal glucose levels in newborns may be lower than those later in life, e.g., ~55–65 mg/dL (~3.05–3.61 mmol/L) in first 24–48 h, which in next days should increase to over 70 mg/dL (3.89 mmol/L) [6]. The child had to be placed in an incubator and managed with intravenous glucose infusions and oxygen therapy. In addition, during the hospitalization, prolonged jaundice was noticed. At the 7th day of life, her level of total bilirubin was 14.9 mg/dL (254.79 µmol/L), which is close to the upper limit of the normal range (15 mg/dL, 256.5 µmol/L), so phototherapy was initiated. Simultaneously, a significant decrease in her body weight was observed; the lowest weight (2770 g) was on her 7th day of life. Later, during the child’s stay at home, the girl was fed with modified milk and ate small amounts of food (30–40 mL), fell asleep while eating and slept a lot. Despite being fed eight times a day, her weight gain was very poor.

The girl was hospitalized at the age of 4 weeks due to prolonged jaundice, a total bilirubin level of 9.5 mg/dL (162.7 µmol/L, direct and indirect bilirubin concentrations not provided) and pneumonia; and in the 2nd month of life due to iron deficiency anemia. The available documentation from both hospitalizations did not include the results of blood glucose measurements; there was also no annotation regarding the occurrence of hypoglycemia. The child’s weight during the hospitalizations was 3000–3100 g, and no significant progress between them.

In the 3rd month of life, the child was admitted to the Hematology Outpatient Clinic of University Pediatric Center, Medical University of Lodz, Poland, due to anemia. Apart from the appropriate hematological assessment, her glucose level was measured, which was 28 mg/dL (1.56 mmol/L); the second result was 24 mg/dL (1.33 mmol/L); the child presented with no clinical signs of hypoglycemia. The girl was immediately admitted to the Department of Pediatric Allergology, Gastroenterology and Nutrition of the same hospital, and after first hormonal test she was transferred to the Department of Pediatrics, Endocrinology, Diabetology and Nephrology. She had stable vital signs; no specific symptoms were noticed. Nevertheless, during her 1st day of hospitalization, recurrent hypoglycemia was confirmed, regressing only via intravenous glucose infusion. Her weight was still about 3100 g.

Results of hormonal testing showed an undetectable level of cortisol (0.00 µg/dL, normal range: 5–25) during hypoglycemia and an inappropriately low concentration of ACTH (5.74 pg/mL; normal range: 0–46). GH levels assessed a few times during hypoglycemia were very low (0.21–0.56 ng/mL) with respect to values expected in GH-sufficient children of this age [7,8,9]. Serum insulin-like growth factor-1 (IGF-1) was undetectable (below 15 ng/mL; normal range < 15–272); free thyroxine (FT4) concentration was low (0.62 ng/dL; normal range: 0.85–1.60); TSH was normal (4.884 µIU/mL; normal range: 0.880–5.420), but inadequately low with respect to FT4. Gonadotropins were not assessed due to the patient’s age and sex. Other blood tests showed anemia with a low hematocrit (21.9%, normal range: 28–42) and a low level of hemoglobin (72 g/L; normal range: 94–130), mild hyponatremia (129.8 mmol/L; normal range: 135–145) and mild hyperkalemia (5.43 mmol/L; normal range: 3.5–5.1). Diabetes insipidus was excluded.

**Figure 1:**
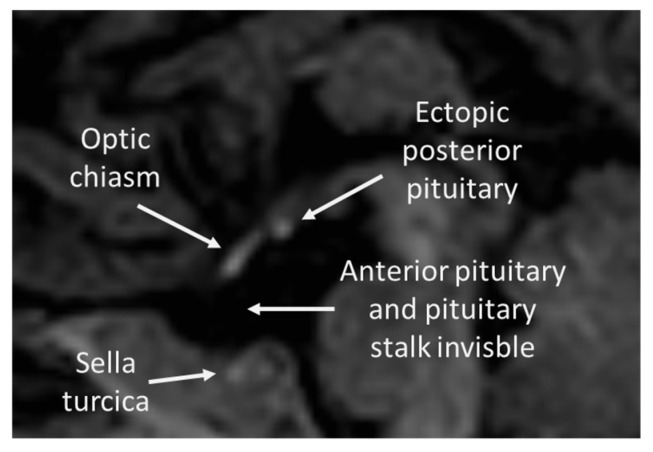
Typical features of PSIS in Patient’s 1 MRI (sagittal plain).

MRI of the brain revealed anterior pituitary aplasia, absent pituitary stalk and ectopic posterior pituitary, which is typical for PSIS (see Figure 1).

The rehabilitation consultation revealed asymmetry in the position of the body and the ranges of limb movements. At the age of 3 months, the girl did not control the position of her head and was unable to keep it in an upright position.

As soon as the diagnosis of CPHD, including severe GHD, secondary adrenal insufficiency and central hypothyroidism, was confirmed, substitution of hydrocortisone at a dose of 3.0 mg three times a day and levothyroxine (LT4) at a dose of 12.5 µg daily was started.

After a few days of hydrocortisone and LT4 substitution, normalization of blood glucose levels was noticed, and the girl no longer needed glucose infusions. Her vital signs were correct. An increase in the amount of food eaten, gradual weight gain and a significant improvement in her general condition were observed. However, initially, supraphysiological doses of hydrocortisone were necessary to maintain normoglycemia. The therapy with recombinant human GH (rhGH) at a dose of 0.25 mg/kg/week (35.7 µg/kg/day) in daily injections (7 days a week) was started few days later (for procedural reasons) and is continued in the Department of Endocrinology and Metabolic Diseases, Polish Mother’s Memorial Hospital—Research Institute (PMMH-RI). After introducing rhGH administration, hydrocortisone doses could be decreased to standard substitutional ones with no occurrence of hypoglycemia. 

At present, the girl is 10 months old, having been treated for over 6 months. Her general condition is good. Motor development has improved significantly—the girl can sit and stand without assistance. Her body weight is 5600 g, and her length is 62 cm. Despite significant improvement in height velocity and height gain of 10 cm over 6 months, her height SDS, calculated according to centile charts of Palczewska and Niedźwiecka for Polish children [10], is still very low and has even decreased (from −4.24 to −4.70). The results of hormonal tests are as follows: cortisol—25.8 µg/dL (normal range: 5–25, sample obtained 2 h after the morning dose of hydrocortisone), FT4—0.73 ng/dL (normal range: 0.92–1.99) and free triiodothyronine (FT3)—3.05 pg/mL (normal range: 2.15–5.83). The serum IGF-1 concentration was 19.5 ng/mL (normal range: 18.7–104.0). Fasting glucose level was 90 mg/dL (5.0 mmol/L). Hormonal substitution is continued—rhGH at the previous dose, hydrocortisone reduced to 2.0 mg three times a day and LT4 at a 50% higher dose. Hormonal substitution is continued simultaneously with intensive rehabilitation.

### 2.2. Patient 2

A girl, a daughter of healthy and not related parents, was born at the 40th week of gestation by vaginal delivery. Her birth weight and length were 4000 g and 54 cm, respectively; her birth length SDS for GA was +1.04; her birth weight SDS for GA was +1.97, according to Niklasson et al. [5]; her Apgar score at birth was 2/7/7/8.

The girl had congenital pneumonia and required respiratory resuscitation, followed by passive oxygen therapy and infant flow breathing support. The physical examination revealed facial dysmorphia—hypertelorism, on a small facial part of the cranium, prominent frontal tubers, palpebral fissures asymmetry and a drooping right upper eyelid. Hypoglycemia with the lowest glucose level of 32 mg/dL (1.77 mmol/L), requiring intravenous glucose administration, was observed in the neonatal period, together with prolonged jaundice and hyperbilirubinemia (unfortunately, the information concerning the exact duration of hypoglycemia and the highest bilirubin level is missing).

At 6 months of age, the girl was hospitalized in the Pediatric Department of the district hospital due to a respiratory tract infection. Laboratory tests showed low levels of FT4 and FT3, and TSH was found to be slightly elevated (6.2 µIU/mL; normal range not provided in documentation). As hypothyroidism was diagnosed, LT4 substitution at a dose of 25 µg/day was initiated, and the girl was discharged from the hospital. In control blood tests, pharmacological euthyroid state was confirmed. 

At the age of 10 months, the child was hospitalized in the Department of Neurology due to psychomotor retardation. MRI of the brain revealed hypoplastic sella turcica, anterior pituitary aplasia, an absent pituitary stalk and ectopic posterior pituitary tissue (typical features of PSIS). Then, due to MRI abnormalities, the girl was hospitalized for the first time in the Department of Endocrinology in one of the provincial cities. Laboratory tests showed a normal glucose level and normal serum electrolytes. Results of hormone testing revealed normal levels of TSH, FT4 and FT3 (1.038 µIU/mL, 0.77 ng/dL and 2.87 pg/mL, respectively; normal ranges not provided, but the results were labelled normal). The concentration of cortisol was low (4.2 µg/dL; normal range: 5–25 µg/dL). Thus, adrenal insufficiency was suspected and a short Synacthen test (SST) performed. In this test, the level of cortisol increased up to 14.3 µg/dL (395 nmol/L), which was labelled as confirming the normal adrenal reserve. It should be noted that the correct response to stimulation in SST is an increase in cortisol level to above 18 µg/dL (500 nmol/L) or 19.9 µg/dL (550 nmol/L) [11]. During this hospitalization, only the dose of LT4 was increased up to 37.5 µg/day, and hydrocortisone replacement therapy was not administered. Unfortunately, the function of somatotrophic axis was not assessed. Instead, the assumption of intrauterine vitamin D deficiency was made due to the appearance of the child (i.e., prominent frontal tubers).

During the 2nd year of life, the girl was hospitalized many times in the Pediatrics, Immunology and Nephrology Department, PMMH-RI, due to infections with accompanying vomiting and high fever. Recurrent hypoglycemia with the lowest glucose level of 45 mg/dL (2.5 mmol/L) and severe hyponatremia (110–112 mmol/L) were also observed at that time. For this reason, at the age of 2 years the girl was admitted to the Department of Endocrinology and Metabolic Diseases, PMMH-RI. The physical examination revealed features of craniofacial dysmorphia, weight and height deficiency and delay in psychomotor development (she could sit and stand without support but was unable to walk; her speech development was also delayed). At the age of 26 months her height was 78 cm, making her height SDS −3.85 according to centile charts of Palczewska and Niedźwiecka [10], and weight was 10.4 kg (appropriate for her height age). Low cortisol levels in the morning (1.15 ug/dL; normal range: 6.2–19.4) and in the afternoon (0.76 µg/dL; normal range: 2.3–11.8), and inadequate ACTH levels (17.6 pg/mL and 11.2 pg/mL, respectively; normal range for morning hours: 0–46), were observed. The test of GH secretion after falling asleep was also done; the GH peak was only 0.19 ng/mL (in Poland this test was the latest in 2022—a screening procedure for diagnosing GHD in children, with the cut-off value of GH peak 10.0 ng/mL). Due to the patient’s age being below 2 years, GH stimulation tests with pharmacological agents were not performed. Secondary IGF-1 deficiency was confirmed by undetectable IGF-1 concentration (below 25 ng/mL; normal range: 49–289), and IGF binding protein-3 (IGFBP-3) was also low (0.94 µg/mL; normal range: 0.9–4.3). The patient’s bone age was assessed at 1–1.5 years, according to Greulich-Pyle standards [12]. The girl had the normal female karyotype, 46,XX. 

The patient was diagnosed with CPHD, including severe GHD, secondary adrenal insufficiency and central hypothyroidism. Treatment with hydrocortisone at a dose of 3.0 mg two times a day was started and the substitution of LT4 at a dose of 37.5 µg once a day were continued. Due to persistent hyponatremia, the girl required additionally a small dose of fludrocortisone (0.025 mg/day) that is typically necessary in patients with primary but not secondary adrenal insufficiency. After putting the child in the Therapeutic Program, rhGH therapy was started at a dose of 0.20 mg/kg/week (29 µg/kg/day) in daily injections (7 days a week). 

After 3 months of rhGH therapy, the patient’s general condition improved. The girl began to walk without assistance, and her speech development clearly accelerated. The concentration of IGF-1 was normal (75.1 ng/mL; normal range: 49.0–289.0), and so was that of IGFBP-3 (3.05 µg/mL; normal range: 0.90–4.00). During first 10 months of treatment, a catch-up of growth by 14.0 cm was observed; her height SDS increased to −1.11.

In the subsequent years, the therapy with rhGH, hydrocortisone, fludrocortisone and LT4 was continued in the doses adjusted to patient’s weight or body area and to the results of the control hormonal test. 

At present she is 9.7 years old; rhGH therapy duration has been 7.5 years. The girl’s height is 143.5 cm; her height SDS is +0.98; her bone age assessed at the age of 9.0 years corresponds to the Greulich–Pyle standard for 8 years 10 months [12]. 

**Figure 2:**
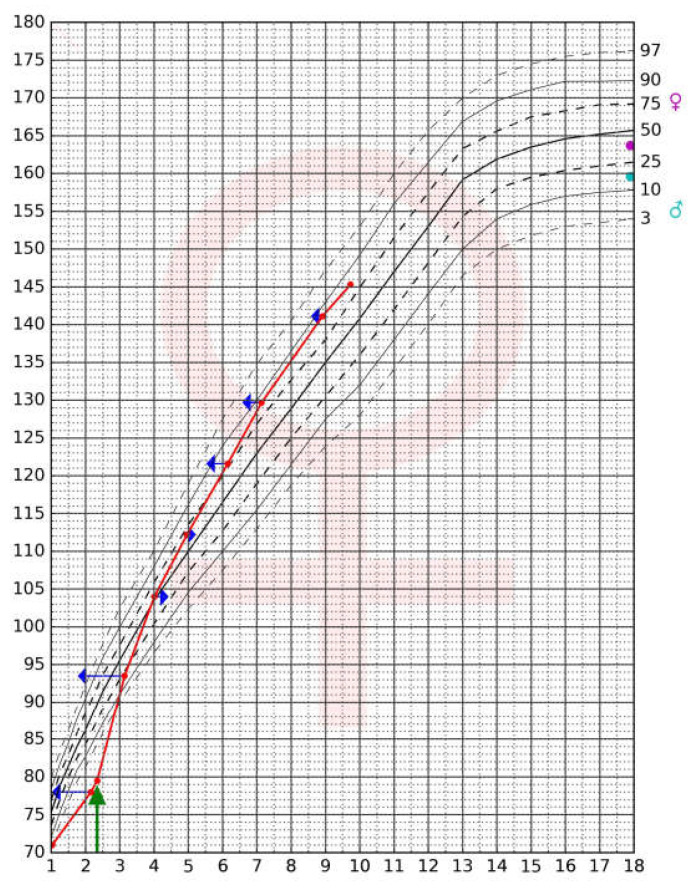
Growth curve of Patient 2 before and during rhGH therapy. Patient’s growth curve on the centile chart for girls of Palczewska and Niedźwiecka [10]: horizontal axis—patient’s age (years), vertical axis—patient’s height (cm); dark blue arrows—bone age of the girl at particular time points, assessed according to Greulich-Pyle standards [12]; green arrow—rhGH therapy onset; pink dot—mother’s height, blue dot—father’s height SDS transposed into a centile chart for girls.

The last laboratory tests results were normal: TSH—0.98 µIU/mL (normal range: 0.60–4.84), FT4—1.04 ng/dL (normal range: 0.97–1.67), IGF-1—367.8 ng/mL (normal range: 112.0–398.0) and IGFBP-3—4916 µg/mL (normal range: 2951–6476). Glucose, sodium and potassium levels were normal. Hormonal substitution was continued: rhGH in a dose of 0.175 mg/kg/week (25 µg/kg/day) in daily injections (7 days a week), hydrocortisone at 5.0 mg two times a day, fludrocortisone at 0.025 mg/day and LT4 in one daily dose of 75 µg. During the substitution therapy, the patient had no hypoglycemia or hyponatremia; there were no adverse events that could be related to treatment. According to current guidelines, in the absence of puberty, assessment of gonadotropin secretion in a test with GnRH should be performed at the age of 10–11 years, and sex steroid substitution should be introduced in cases of confirmed hypogonadotropic hypogonadism (that seems highly probable in our patient) [13]. The growth curve of the Patient 2 is presented in Figure 2.

## 3. Literature Review

As the symptoms of CPHD vary among the patients and may appear in different configurations, we have compared our patients with other cases that were described in the literature to find similarities and differences. 

Mehta and Brar [6] described five patients with persistent neonatal hypoglycemia. All children were full term and born by cesarean section; all had bradycardia and apnea in the neonatal period; all required glucose infusions due to hypoglycemia in first days of life. In four of them, the diagnosis of CPHD was established in the first days of life; however, in one case the diagnosis was delayed, as hypoglycemia persisting during first 11 days of life had been considered as transient, and the child was readmitted to the hospital at the age of 52 days due to hypoglycemic seizures. Hydrocortisone and rhGH substitution was necessary to maintain normoglycemia and to withdraw glucose infusions. All children had secondary hypothyroidism and required LT4 supplementation; none of them had diabetes insipidus. After MRI, all of them were diagnosed with PSIS, and there was septo-optic dysplasia (SOD) in two cases. All three male patients from that study had micropenises. One child was diagnosed with cholestasis, which was considered by the authors as related to the late diagnosis of CPHD and delayed onset of treatment with hydrocortisone and LT4. 

Cavarzere et al. [14] also reported five cases of children with congenital hypopituitarism. All of them were born at term, four by vaginal delivery, one by cesarean section. All had neonatal hypoglycemia that in two of them was observed also after the neonatal period; four also had jaundice (in two cases assessed as cholestatic and in two as neonatal). Only those two children with cholestatic jaundice were properly diagnosed as newborns; for the remaining three the diagnosis was established at the age of 2–8 years, when they presented with severe short stature. Finally, four children were diagnosed with CPHD. The only child who presented without jaundice was diagnosed with IGHD. None of the patients had diabetes insipidus. MRI showed all the components of PSIS in four of them; one child had also Chiari malformation. All five patients required substitution of rhGH; four children additionally received hydrocortisone; and two received LT4. Clinical signs, results of laboratory tests and results of hypothalamic-pituitary imaging for the five children were similar to our cases. 

Chen et al. [15] described a girl with congenital hypopituitarism due to *POU1F1* gene mutation. She was born at term by spontaneous vaginal delivery. A few days after birth, hypothermia and hypoglycemia were observed. At that time, secondary hypothyroidism was diagnosed, and the girl received LT4 substitution. At the age of 8 months, poor body weight and length gain, motor development retardation and midline abnormalities (e.g., saddle nose) were noticed. Hormonal tests confirmed secondary hypothyroidism and led to the diagnosis of severe GHD and hypoprolactinemia; concentrations of ACTH and cortisol were normal. MRI of the brain revealed anterior pituitary hypoplasia and an abnormal pituitary stalk. The symptoms and deficiencies of TSH, GH and Prl perfectly reflected the clinical picture of CPHD caused by a mutation in *POU1F1* gene. Subsequently, a novel mutation of this gene was identified in the patient by whole exome sequencing (WES).

Other case reports concern patients with PSIS, in whom neonatal hypoglycemia did not occur or was not considered the important question, and was diagnosed later due to growth failure.

Boros et al. [16] described seven patients with PSIS, diagnosed at the age from 10.5 to 27.0 years, with normal perinatal history, in whom the first problem was short stature, followed by the lack of spontaneous pubertal development in older patients. The authors pointed out the fact that all these patients were either migrants (five cases) or from families with low socio-economic status (two cases).

In 2021, Chrzanowska et al. [17] presented a group of 31 Polish children with radiologically confirmed PSIS. Among them, 74.2% had CPHD, whereas 25.8% presented with IGHD. Age at diagnosis ranged from 0.2 to 16.1 years. Hypoglycemia was a cause of admission to the hospital for only four children, whereas 17 were referred to an endocrinologist due to short stature. Similarly to other cohorts, low birth weight was a rare condition (two cases), and 18 children were born by cesarian section. A history of hypoglycemia in childhood was reported in five cases, and a history of hyperbilirubinemia and cholestatic jaundice in the neonatal period, in three. The diagnosis of GHD was established in all but one patient.

Very recently, Hietamäki et al. [18] have analyzed a cohort of 124 patients with CPHD, treated in a single tertiary center during over 30 years, and identified 48 cases of congenital CPHD, thereby estimating its incidence in the population as 1:16,000. Among these patients, 23 presented with typical features in the neonatal period (severe hypoglycemia; prolonged jaundice; in boys also micropenis or bilateral cryptorchidism); in 13 other patients, the neonatal phenotype was difficult to interpret due to prematurity or missing data. In five cases, the first endocrine investigation was performed after 1 year of life. The boys with micropenises or cryptorchidism were diagnosed earlier, and the delay in diagnosis concerned children with isolated hypoglycemia or jaundice in the neonatal period. In 22 patients, a brain MRI was performed, and a classic form of PSIS was identified in 14 cases, and other combinations of its components in seven ones; extra-pituitary disorders were found in 15 cases, most frequently SOD. Final genetic diagnosis was established in seven patients; however, rare variants of different genes related to CPHD or isolated GHD (mainly of uncertain significance) were found in 18 out of 21 patients subjected to genetic analysis, including WES.

Sertedaki et al. [19] presented two male patients with CPHD and typical symptoms of PSIS in MRI. In each patient, three different heterozygous variants of genes involved in head, hypothalamus and/or pituitary development were identified in WES. The authors have stated that CPHD seems to be an oligogenic disease and that there is a need for further development of the base of genes involved in CPHD, using WES or even whole genome sequencing.

In another recent study, concerning a cohort of patients with PSIS, in whom WES was performed, Brauner et al. [3] demonstrated a complex genetic heterogeneity of PSIS, reflected by phenotypic heterogeneity of the affected patients. In 39 out of 52 patients, they managed to identify novel or rare variants of genes involved in pituitary development or function, midline defects, syndromes related to hypogonadotropic hypogonadism or short stature, optical anomalies, cerebellum atrophy, corpus callosum agenesis and disordered axonal migration.

## 4. Discussion

### 4.1. Hypoglycemia in Newborns and Infants

During pregnancy, maternal blood is a source of continuous glucose supply for the fetus. After birth, glucose homeostasis has to be maintained through intermittent food intake. Normoglycemia is necessary for proper function and maturation of the central nervous system, as glucose is the main source of energy for the brain. In children, metabolic rates of glucose in the brain reach 71–93% of these observed in adults at the age of 5 weeks, and become similar to adult levels from the age of 2 years. Peak blood flow and energy metabolism in the brain, related to its maturation, occurs between 3 and 8 years of life [20]. Glucose uptake by the brain is independent from insulin and is mediated by the family of glucose transporters (GLUT) that are expressed in different tissues. Among them, GLUT1 is located in the brain–blood barrier, and GLUT3 plays the main role in glucose transport to neurons [21,22]. Glucose concentrations in the brain are lower than in the plasma.

The mechanisms of brain adaptation to low circulating glucose concentrations include an increase in cerebral blood flow and—in chronic hypoglycemia—an increase in the number of glucose transporter sites. That allows one to maintain normal cerebral functional activity during moderate acute hypoglycemia; however, severe and prolonged hypoglycemia may be a cause of neuronal damage [20]. At plasma glucose concentrations below 36 mg/dL (2.0 mmol/L), cerebral glucose metabolism exceeds the capacity of glucose transport to the brain [23].

A key role in the regulation of glucose storage and release is the balance between insulin and counterregulatory hormones—glucagon, cortisol, epinephrine and GH. Insulin secretion decreases at glucose concentrations below 80–85 mg/dL (4.4–4.7 mmol/L) and becomes inhibited at glucose levels of 45–54 mg/dL (2.5–3.0 mmol/L). Glucagon, epinephrine, cortisol and GH secretion are activated when glucoses decrease below 65–70 mg/dL (3.5–3.9 mmol/L). In healthy newborns, increases in epinephrine, norepinephrine and glucagon concentrations, together with a decrease of insulin secretion, are observed directly after birth. Excessive insulin secretion, as well as cortisol or GH deficiency, may cause severe and persistent hypoglycemia [24]. During the first 12 h of life, the mechanisms of gluconeogenesis and of ketone generation are immature, contributing to hypoglycemia in the first day of life [25]. Congenital defects in glycogenolysis, gluconeogenesis, fatty acid oxidation or ketogenesis may also cause severe and prolonged neonatal hypoglycemia, which should be considered in differential diagnosis.

In patients with CPHD, persistent and recurrent hypoglycemia during the neonatal period and beyond is related to secondary adrenal insufficiency and severe GHD.

### 4.2. Clinical Picture of Congenital CPHD

Our patients presented with the most typical features of CPHD, including persistent neonatal hypoglycemia, which—unfortunately—was downplayed in that time, leading to a delay in establishing the diagnosis and starting hormonal substitution. Both girls had abnormal MRI of the pituitary region, corresponding to the diagnosis of PSIS. Up to now, the patients have had no genetic defects confirmed; nevertheless, it should be mentioned that for most cases of PSIS, the genetic background of the syndrome is not established.

Among the discussed clinical cases, all children with persistent hypoglycemia had congenital hypopituitarism—in most cases, CPHD. This allows us to create a list of the most common symptoms of CPHD, such as persistent neonatal hypoglycemia (in some cases asymptomatic), lethargy, temperature instability, apnea, severe hyponatremia, prolonged jaundice and failure to thrive; in addition, micropenis and undescended testes in males [26]. Some of these symptoms may be downplayed, such as hypoglycemia in our (but not only our) patients, resulting in the considerable delay of diagnosis. Additionally, non-endocrine clinical manifestations such as midline brain abnormalities, optic nerve hypoplasia [27] and many others may be observed. Birth weight and birth length are usually normal [1].

Different symptoms can be attributed to the deficits of particular hormones. Thus, GHD may present as persistent hypoglycemia in the neonatal period. Later its main symptom is short stature. ACTH deficiency may cause severe hypoglycemia, cholestasis in the neonatal period (as one of cortisol’s functions is increasing bile flow) and poor weight gain [9,28]. TSH deficiency may cause prolonged jaundice and increased sleepiness in the neonatal period, along with other typical symptoms of hypothyroidism later in life [9]. Gonadotropin deficiency leads to hypogenitalism in male infants and hypogonadotropic hypogonadism in both sexes that may be identified starting from the age appropriate for puberty. Some other phenotype features typical for particular genetic defects may occur.

Severe GHD is a common component of CPGD. Early rhGH substitution is important not only for correcting hypoglycemia and maintaining normal linear growth, but also for improvement of psychomotor development, as has been observed in our patients. Similar beneficial effects of rhGH administration have previously been reported in the studies on rats after experimental brain injuries (recovery of lost motor function) [29], as well as humans [30,31].

### 4.3. Pleiotropic Effects of GH and Consequences of GHD

Apart from stimulation of human growth and cell proliferation, GH is one of the most important hormones involved in the regulation of metabolic processes. Some of the effects of GH are direct, whereas others are mediated by IGF-1.

GH plays an important role in glucose homeostasis, especially supporting glucose levels in terms of prolonged fasting, and has a less pronounced effect on insulin-induced hypoglycemia. This effect is mediated by limiting insulin sensitivity, reducing glucose utilization and increasing lipolysis [32,33,34]. The effect of GH on carbohydrate metabolism is complex, as directly it works antagonistically to insulin as one of counterregulatory hormones, and indirectly—via IGF-1—it has an insulin-like effect [34,35,36]. A GH-mediated increase in glucose concentration is related to an increase of gluconeogenesis in the liver, together with a decrease in glucose transport to peripheral tissues and glucose oxidation.

In adipose tissue, GH stimulates lipolysis, leading to the increased release of free fatty acids and glycerol into the blood stream. Additionally, GH intensifies lipid oxidation, which enhances the formation of ketones [34]. In patients with GHD, an atherogenic lipid profile is observed together with increased body fay mass and an increase in blood pressure [37]. Therapy with rhGH therapy in GH-deficient patients leads to improvement of the lipid and adipokine profile, and body composition (a decrease of fat mass) [38,39]. These GH effects are mediated by IGF-1; however, metabolic pathways and molecular mechanisms related to these processes require clarification in further studies [39].

GH’s anabolic effects on protein metabolism include an increase in protein synthesis and a decrease in their degradation, along with a reduction in urea synthesis in the liver. These effects are significant, especially during exercise and starvation, and are less pronounced in basal state. Both protein synthesis and breakdown are reduced in GHD. The anabolic effect of GH is important for the psychomotor development of children, as it increases muscle mass and strength [36].

The effects of GH on the heart are complex and include sustaining myocardial architecture, preserving its capillary density, increasing mechanical stretching of heart muscle and anti-ischemic reperfusion. GHD and low levels of IGF-1 are associated with structural and functional cardiac abnormalities that may be at least partially restored during rhGH therapy. These issues have recently been widely discussed by Napoli et al. [40].

The main effect of GH on bones is stimulating their linear growth and maturity, alongside increasing bone mineral density and bone mineral content. These effects are related to the acceleration of bone turnover by direct stimulation of proliferation and activity of osteoblasts (bone formation) and by increasing the number and differentiation of osteoclasts (bone resorption), with the predominance of bone formation. Indirect effects of GH on bones are mediated by IGF-1 derived from hepatic synthesis, and produced locally by osteoblasts and acting in a paracrine or autocrine manner. IGFBP-3 acts synergically with IGF-1, enhancing its effect on osteoblast proliferation and collagen synthesis [41,42]. Other GH effects include increasing the synthesis of an active form of vitamin D—1.25 (OH)_2_D_3_—by activating 1α-hydroxylase in kidneys and enhancing sex steroid secretion and its effects on bone tissue. These issues have been discussed in detail by Esposito et al. [43]. Accordingly, GHD leads to reduced bone turnover and bone mass [44]. In children with GHD, rhGH substitution improves adult height, increases bone mineral density and helps one to obtain an optimal peak bone mass; however, a small decrease in bone mineral density in the initial phase of treatment should be taken into account [45].

GH effects gonadal function on several levels. It stimulates gonadotropin secretion from the pituitary gland, along with the sensitivity of gonads to gonadotropins, thereby enhancing the production of sex steroids [46]. In women with panhypopituitarism, rhGH substitution—in addition to gonadotropins, LT4 and hydrocortisone—may be essential for obtaining pregnancy [47]. Current knowledge on GH’s influence on ovaries has recently been discussed by Devesa and Caicedo [48], and interactions between GH/IGF-1 and testicular function by Tenuta et al. [49]. However, GH secreted by the pituitary gland of the fetus seems to have no evident effects on intrauterine growth, as the birth sizes of newborns with congenital GHD are, in general, within the normal range [1,8,9,18].

### 4.4. Human Brain as a Target Organ for GH

In recent years, GH-responsive neurons have been identified in different areas of the brain, especially in hypothalamic nuclei. These findings have highlighted the role of GH in the central regulation of appetite, energy expenditure, glucose homeostasis and neuroendocrine changes. While GH is secreted in a pulsatile manner, some of its effects are achieved indirectly, via IGF-1, whose concentration and bioavailability are more stable. Apart from GH and IGF-1 concentrations, the sensitivity of their receptors plays an important role in signal transmission to target cells and tissues.

The widespread presence of GH-responsive neurons in different areas of the brain has been documented, mainly in animal studies, suggesting that GH may directly modulate different neural functions [50,51]. Among others, GH plays a role in food intake regulation and in maintaining blood glucose concentrations during prolonged food restriction. The role of GH action in the central regulation of metabolic processes has recently been summarized by Donato et al. [51]. An important player linking stimulation of GH secretion and food intake is ghrelin. The interactions among GH, IGF-1 and ghrelin, especially in the context of GHD, have been summarized by Lewinski et al. [52], and the influence of orexigenic and satiety hormones (ghrelin and nesfatins, respectively) on pituitary GH secretion, by Devesa [53].

An interesting issue is GH’s contributions to cell proliferation and recovery of lost motor functions after brain injuries or cortical ablation, documented in the studies on rats [29,54]. The therapy with rhGH may contribute to recovery in children with cerebral palsy [30], after asphyxia during delivery [31], after traumatic brain injury [55] or with congenital defects of spinal cord development (caudal regression syndrome) [56]. The same mechanisms may be important in children with acquired GHD and should be considered in patients with delayed diagnosis of congenital IGHD or CPHD.

### 4.5. Genetic Causes of CPHD

Etiology of non-syndromic CPHD includes sporadic or familiar mutations in two genes, *PROP1* and *POU1F1*, which cause dysfunction of the anterior pituitary (see Table 1) [57]. Mutations of *PROP1* are the most common genetic causes of hypopituitarism in humans [58]. In most patients, the first symptom is IGHD, which presents early in the life. TSH, ACTH and gonadotropins deficiencies are highly variable in terms of the onset and severity of symptoms [28,59]. The human *POU1F1* gene is involved in the regulation of genes encoding GH, TSH and Prl. ACTH and gonadotropins are usually normal [57]. In general, GH and Prl deficiencies appear soon after birth, whereas the onset of symptoms of TSH deficiency occurs later. However, the range of deficiencies of these hormones varies significantly among the patients with *POU1F1* mutations [58].

As previously mentioned, CPHD can appear in association with morphological defects of the craniofacial and forebrain midline [4,28]. Genetic disorders related to syndromic CPHD are summarized in Table 2.

As the phenotypes of particular syndromes differ significantly from each other, and in some cases genetic diagnosis is not established, impacts from other genetic and environmental factors seem possible. New genome sequencing methods have identified, and should identify in future, other genes involved in CPHD.

### 4.6. Diagnosis of CPHD

Early diagnosis of CPHD can be difficult in neonates because of many factors, such as immature functioning of the hypothalamic–pituitary–adrenal (HPA) axis, prematurity and other complications of the neonatal period. Hypoglycemia in the first days of life is a common metabolic disorder, and it usually does not cause concern among physicians [6]. In healthy newborns during first 48 h of life, hypoglycemia may be caused by a lower glucose threshold for suppression of insulin secretion than later in life, together with immaturity of the enzymes involved in gluconeogenesis [60]. Hypoglycemia that occurs after the 3rd day of life or persists for more than 3 days is referred to as persistent hypoglycemia.

Diagnosis of CPHD includes documenting deficiencies of particular anterior pituitary hormones and secondary insufficiencies of peripheral endocrine glands.

The diagnosis of secondary adrenal insufficiency is based on decreased cortisol concentrations with inappropriately low ACTH levels. Tests that can be performed include multiple random cortisol measurements (low concentrations in all samples, but levels of cortisol are usually lower in neonates in whom, additionally, the circadian pattern of cortisol secretion is usually absent). Low cortisol levels during hypoglycemia have too low specificity that rot the diagnosis of ACTH deficiency [9]. The standard test with Synacthen is safe in infancy, but its protocol has not been well assessed in infants; its sensitivity is about 80%. Nevertheless, a normal result in this test does not rule out ACTH deficiency [61]. In the patients with ACTH deficiency, aldosterone secretion is not affected.

Diagnostic criteria of secondary hypothyroidism include decreased FT4 concentration, together with TSH level either decreased or within a normal range, or even slightly elevated but inappropriately low [9]. However, in neonates born prematurely or with other severe health problems, the euthyroid sick syndrome presenting also with decreased TSH and FT4 levels should be taken into account.

In diagnosing GHD in the youngest children, standard GH stimulation tests are contraindicated [9]. Thus, GHD may be confirmed by low (usually very low) GH concentrations in blood samples obtained in terms of hypoglycemia. According to the current guidelines of Grimberg et al. [7], GHD may be diagnosed without performing GH stimulation tests in children fulfilling three criteria: (1) auxological defect (short stature), (2) hypothalamic-pituitary defect (including features of PSIS, tumors or irradiation) and (3) confirmed deficiency of at least one other anterior pituitary hormone. Congenital GHD can be diagnosed without GH stimulation tests in newborns with hypoglycemia connected with serum GH concentrations below 5 µg/L in whom a deficiency of at least one additional pituitary hormone and/or typical features of PSIS in MRI are observed. However, a low GH concentration during hypoglycemia is not sufficient as the only criterion for the diagnosis of GHD [7]. Binder at al. [62] stated that in newborns, random GH concentrations below 7 µg/L, measured with Mediagnost ELISA kits, are diagnostic for GHD, and for IMMULITE 2000 assays, the recalculated cut-off level should be 9.7 µg/L. The physiologically high GH secretion in the first days of life decreases rapidly during the following weeks [3], which may cause difficulties in children in whom neonatal hypoglycemia did not occur, was overlooked or was downplayed. In addition, IGF-1 and IGFBP-3 are low in such children; however, in the youngest age groups, the lower limit of the normal range of IGF-1 may be below the sensitivity of the assay. Auxological assessment, especially confirming short stature, is not a question in newborns and infants, as the birth size of children with CPHD is usually normal [1,8,9,18].

The diagnosis of gonadotropin deficiency in infants may be challenging. Low basal LH and FSH concentrations are physiological at this age; however, mini-puberty should occur between 15 days and 6 months of life, in boys with a testosterone peak between 4 and 10 weeks. In boys, LH concentrations below 0.8 IU/L, together with testosterone below 30 ng/mL between 5 days and 6 months of life, speak for gonadotropin deficiency. The same is true for FSH concentrations below 0.1 IU/L between 5 days and 2 years of life in girls [12]. The interpretation of a stimulation test with gonadoliberin (GnRH) should be different during mini-puberty and later (absent gonadotropin response before the age of 18 months may contribute to the diagnosis). In boys, a stimulation test with human chorionic gonadotropin (hCG) shows a poor testosterone response [26,27].

If hypopituitarism is suspected or diagnosed, MRI of the brain and hypothalamic-pituitary area should be performed. Abnormalities to look for include anterior pituitary hypoplasia/aplasia/hyperplasia, ectopic posterior pituitary, absent pituitary stalk and other midline forebrain defects [2].

Patients with PSIS have multiple anterior pituitary hormone deficiency or IGHD, but ADH deficiency is uncommon. However, clinical manifestations of PSIS may be different, from persistent neonatal hypoglycemia to jaundice, suggesting CPHD and growth retardation later in life.

A genetic background of PSIS has previously been identified in only 5% of cases, but new genome sequencing methods seem to be of particular value in revealing genetic causes of PSIS and CPHD. Thus, genetic assessment should be a part of diagnostics in such patients.

### 4.7. Hormonal Therapy of CPHD

The therapy of CPHD includes two main directions: symptomatic treatment (immediate correction of hypoglycemia and hyponatremia) and hormonal substitution (hydrocortisone, LT4, GH and sex hormones).

Patients with secondary adrenal insufficiency need to be treated with hydrocortisone or cortisone acetate. Substitution must be started just after diagnosis to prevent an adrenal crisis. The aim of treatment is to mimic a physiological pattern of cortisol secretion: higher doses in the morning and lower in the afternoon [10]. The initial daily dose of hydrocortisone should be 9–12 mg/m^2^, divided into 3 or 4 doses. It is very important to increase the dose of hydrocortisone (twice or thrice) during illness, fever, physical exercise or stress, and to give even higher doses intravenously in cases of emergency [9].

In patients with central hypothyroidism, treatment with LT4 should be initiated, but first it is important to establish that the function of the HPA axis is normal, or that hydrocortisone treatment has been started, as LT4 increases hepatic clearance of cortisol. The initial daily dose of LT4 in newborns is 10–15 µg/kg or even higher in children with malabsorption (that refers, e.g., to ones with cholestasis) [9].

Administration of rhGH is necessary regardless of the child’s growth due to metabolic indications, mainly to prevent hypoglycemia. It is recommended to start from the dose of 0.16–0.24 mg/kg/week. During rhGH therapy, the function of thyroid and adrenal glands should be monitored because GH replacement may unmask hypothyroidism and/or adrenal insufficiency [9].

Sex steroid therapy should be started at the age appropriate for puberty. In male infants with micropenis, testosterone may be also administered in the first months of life, optimally between 1 and 6 months (25 mg of testosterone cypionate or enanthate every 4 weeks for 3 months) [9]. In addition, gonadotropins may be useful, especially for obtaining fertility. Details of these issues go beyond the scope of the paper.

Hormonal substitution should be monitored and adjusted according to treatment standards. An important focus for patients with hypopituitarism is regular screening for manifestation of other pituitary hormone deficiencies, but also for detection of consequences of hypopituitarism [28].

## 5. Conclusions

Taking into account a relatively high incidence of delayed diagnosis of CPHD, even in cases of typical symptoms in the neonatal period, pediatricians and neonatologists need to be watchful when a child develops such symptoms. Hormonal tests and brain MRI should be performed as soon as possible, because delayed diagnosis and lack of appropriate treatment of CPHD can be life-threatening, especially in cases of adrenal insufficiency with undetectable cortisol levels and/or severe GHD leading to persistent and recurrent hypoglycemia. Appropriate hormonal substitution, even delayed, significantly improves somatic and psychomotor development of children. The therapy with rhGH is necessary not only for somatic growth, but also, and even above all, for metabolic reasons. Further studies using new genome sequencing seem necessary to explain the complex genetic basis of CPHD related to PSIS.

## Figures and Tables

**Table 1:** Genetic disorders in non-syndromic combined pituitary hormone deficiency [28,59].

Gene	Inheritance	Pituitary Hormone Deficiencies	Phenotype
*PROP1*	AR	GH, TSH, LH, FSH, Prl and ACTH (evolving)	Anterior pituitary hypoplasia or transient hyperplasia
*POU1F1*	AR, AD	GH, TSH, Prl	Anterior pituitary hypoplasia

AR—autosomal recessive, AD—autosomal dominant.

**Table 2:** Genetic disorders in syndromic combined pituitary hormone deficiency (only the syndromes involving GHD are included) [26,28,58,59].

Gene	Inheritance	Pituitary Hormone Deficiencies	Phenotype
*LHX3*	AR	GH, TSH, LH, FSH, Prl, variable ACTH	Short cervical spine, limited head and neck rotation, sensorineural deafness, pituitary hypo- or hyperplasia
*LHX4*	AD	GH, TSH, ACTH, variable LH and FSH	Cerebellar abnormalities, anterior pituitary hypoplasia, with or without ectopic posterior pituitary, small sella turcica
*HESX1*	AD, AR	Isolated GHD or CPHD	Septo-optic dysplasia, hypoplastic or aplastic anterior pituitary, ectopic or undescended posterior pituitary, hypoplastic or absent corpus callosum
*SOX2*	AD	LH, FSH, variable GH	Anophthalmia or microphthalmia, mental retardation, sensorineural hearing loss, esophageal atresia, genital abnormalities, anterior pituitary hypoplasia
*SOX3*	X-linked	Isolated GHD or CPHD	Mental retardation (variable), hypothalamic and infundibular abnormalities, absent or hypoplastic pituitary stalk, ectopic or undescended posterior pituitary
*GLI2*	AD	CPHD (GH, TSH, LH, FSH or ACTH)	Holoprosencephaly, polydactyly, midline defects, ectopic pituitary stalk, corpus callosum agenesis

AR—autosomal recessive, AD—autosomal dominant.
